# Supplementary figures and images for: Rapid and sensitive detection of genome contamination at scale with FCS-GX
Source: bioRxiv. 2023 Jun 6:2023.06.02.543519. Preprint. [Version 1] doi: 10.1101/2023.06.02.543519 (PMC10246020; doi:10.1101/2023.06.02.543519)

Prokaryotes

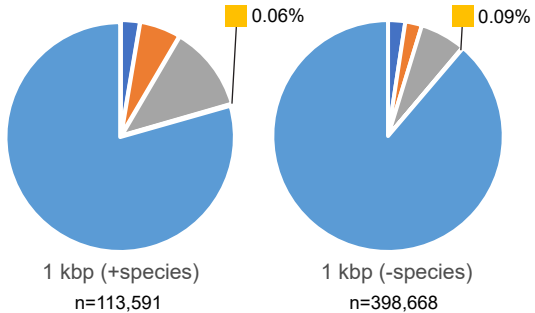

Eukaryotes

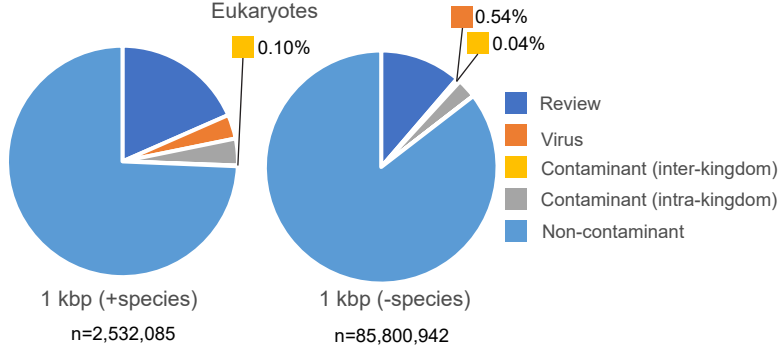

Supplement: Supplement 2 — Fig. S2 Summary of FCS-GX results for false negatives in sensitivity tests. For 1 kbp sequence sets, aggregate counts of false negatives are shown for FCS-GX runs while including the same species tax-ids as the source genome during the alignment stage (+species) and while excluding same species tax-ids (−species). Categories are classified as follows: Review – sequences with the FCS-GX action REVIEW that are assigned the proper contaminant taxonomy but with subthreshold alignment coverage, Virus – sequences assigned prokaryote virus in prokaryote genomes and eukaryote virus in eukaryote genomes, Contaminant (inter-kingdom) – sequences assigned as contaminant by FCS-GX but the taxonomic classification is wrong and is in a different kingdom grouping. Contaminant (intra-kingdom) – sequences assigned as contaminant by FCS-GX but the taxonomic classification is wrong and is the same kingdom grouping. Non-contaminant – sequences assigned as non-contaminant. See Additional file 2: Table S2 for counts/percentages of all false negative categories. [file media-2.pdf]

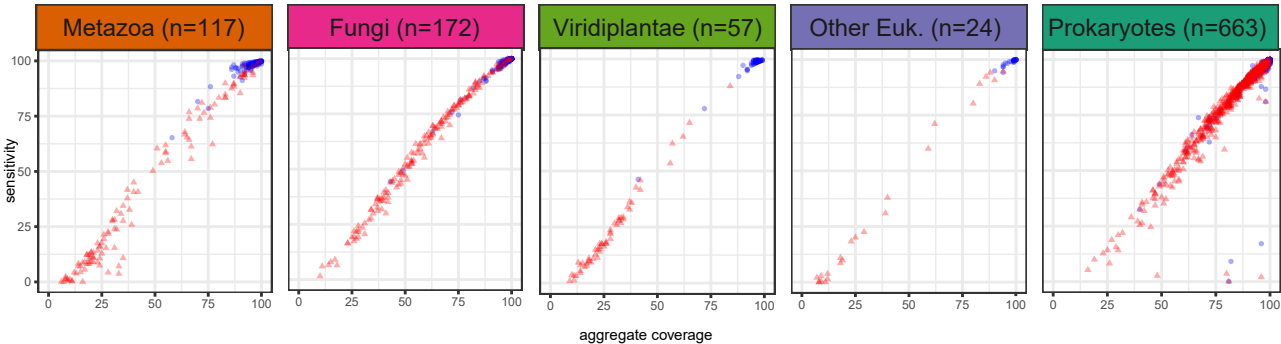

Supplement: Supplement 3 — Fig. S3 Plots of aggregate FCS-GX alignment coverage against sensitivity. Aggregate coverage is calculated as the total percentage of the genome with overlaps from sequences in the FCS-GX reference database. Results are shown for 1 kbp sequence sets for FCS-GX runs while including the same species tax-ids as the source genome during the alignment stage (blue circles) and while excluding same species tax-ids (red triangles). [file media-3.pdf]

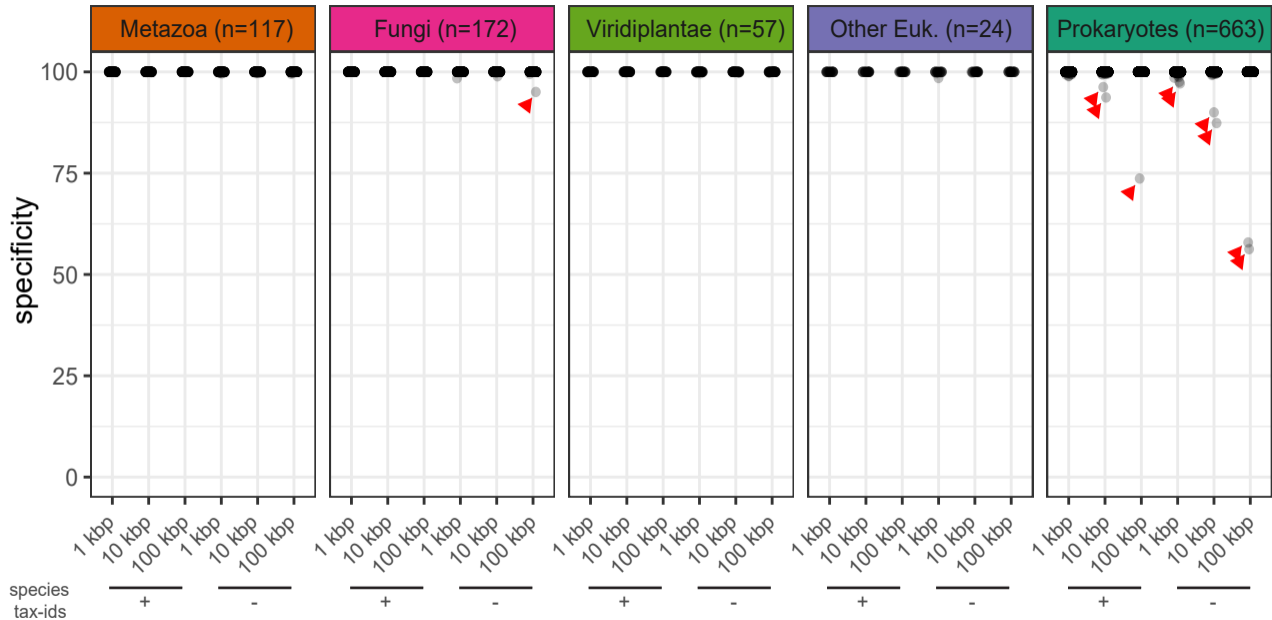

Supplement: Supplement 4 — Fig. S4 Complete distributions of specificity measurements. Distributions are shown for artificially fragmented genomes in five “kingdom” groups. Specificity is shown for genomes fragmented at three different window sizes (1 kbp, 10 kbp, 100 kbp). For each window size, specificity is shown for FCS-GX runs while including the same species tax-ids as the source genome during the alignment stage (+species) and while excluding same species tax-ids (−species). Red arrows point to ten outliers that are not visualized in Fig. 2B. [file media-4.pdf]

frequency (millions of sequences)

15  
10  
5  
0

0

5

10

15

>20

contaminant length (kbp)

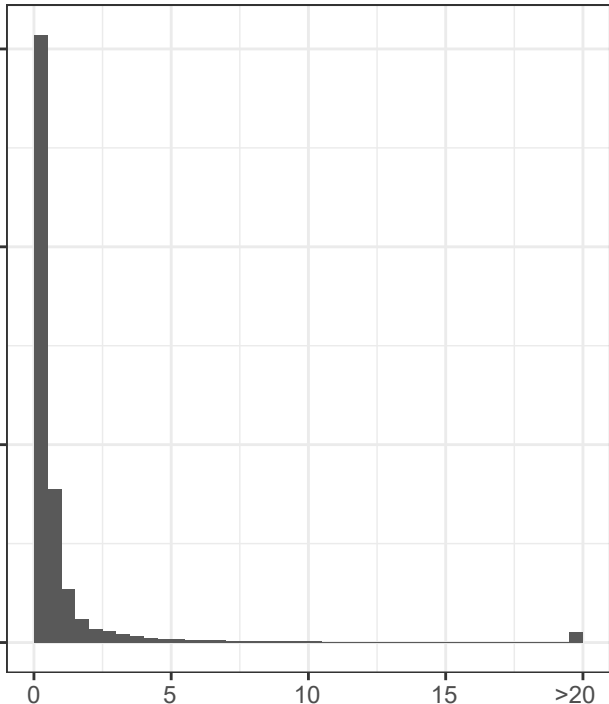

Supplement: Supplement 5 — Fig. S5 Length distribution of contaminants detected by FCS-GX. [file media-5.pdf]
