# Supplementary material for: Rapid and sensitive detection of genome contamination at scale with FCS-GX
Source: bioRxiv. 2023 Jun 6:2023.06.02.543519. Preprint. [Version 1] doi: 10.1101/2023.06.02.543519 (PMC10246020; doi:10.1101/2023.06.02.543519)
Supplement: 1 [file NIHPP2023.06.02.543519V1-supplement-1.pdf]

bioRxiv preprint doi: https://doi.org/10.1101/2023.06.02.543519; this version posted June 6, 2023. The copyright holder for this preprint (which was not certified by peer review) is the author/funder. This article is a US Government work. It is not subject to copyright under 17 USC 105 and is also made available for use under a CC0 license.

Supplementary Information

Additional file 1: supplementary figures

Fig. S1

FCS-GX commands and sample output.

Fig. S2

Summary of FCS-GX results for false negatives in sensitivity tests. For 1 kbp sequence sets, aggregate counts of false negatives are shown for FCS-GX runs while including the same species tax-ids as the source genome during the alignment stage (+species) and while excluding same species tax-ids (-species). Categories are classified as follows: Review – sequences with the FCS-GX action REVIEW that are assigned the proper contaminant taxonomy but with subthreshold alignment coverage, Virus – sequences assigned prokaryote virus in prokaryote genomes and eukaryote virus in eukaryote genomes, Contaminant (inter-kingdom) – sequences assigned as contaminant by FCS-GX but the taxonomic classification is wrong and is in a different kingdom grouping. Contaminant (intra-kingdom) – sequences assigned as contaminant by FCS-GX but the taxonomic classification is wrong and is the same kingdom grouping. Non-contaminant – sequences assigned as non-contaminant. See **Additional file 2: Table S2** for counts/percentages of all false negative categories.

Fig. S3

Plots of aggregate FCS-GX alignment coverage against sensitivity. Aggregate coverage is calculated as the total percentage of the genome with overlaps from sequences in the FCS-GX reference database.

bioRxiv preprint doi: https://doi.org/10.1101/2023.06.02.543519; this version posted June 6, 2023. The copyright holder for this preprint (which was not certified by peer review) is the author/funder. This article is a US Government work. It is not subject to copyright under 17 USC 105 and is also made available for use under a CC0 license.

Results are shown for 1 kbp sequence sets for FCS-GX runs while including the same species tax-ids as the source genome during the alignment stage (blue circles) and while excluding same species tax-ids (red triangles).

Fig. S4

Complete distributions of specificity measurements. Distributions are shown for artificially fragmented genomes in five “kingdom” groups. Specificity is shown for genomes fragmented at three different window sizes (1 kbp, 10 kbp, 100 kbp). For each window size, specificity is shown for FCS-GX runs while including the same species tax-ids as the source genome during the alignment stage (+species) and while excluding same species tax-ids (-species). Red arrows point to ten outliers that are not visualized in **Fig. 2B**.

Fig. S5

Length distribution of contaminants detected by FCS-GX.

bioRxiv preprint doi: https://doi.org/10.1101/2023.06.02.543519; this version posted June 6, 2023. The copyright holder for this preprint (which was not certified by peer review) is the author/funder. This article is a US Government work. It is not subject to copyright under 17 USC 105 and is also made available for use under a CC0 license.

Additional file 2: supplementary tables

Table S1

FCS-GX sensitivity and specificity scores on artificially fragmented genomes.

Table S2

Counts and percentages of FCS-GX false negative types for sensitivity tests on 1 kbp sequences.

Table S3

FCS-GX contamination calls in 100 kbp fragmented genome datasets produced during specificity tests.

Table S4

Upper and lower bound estimates of FCS-GX sequence-level specificity.

Table S5

Aggregate contamination identified in current GenBank genomes by FCS-GX, grouped by taxonomic kingdom.

Table S6

Aggregate contamination identified by FCS-GX contained in GenBank and RefSeq databases from 2017-2023.

Table S7

Aggregate contamination identified in current GenBank genomes by FCS-GX, grouped by FCS-GX taxonomic division.

bioRxiv preprint doi: https://doi.org/10.1101/2023.06.02.543519; this version posted June 6, 2023. The copyright holder for this preprint (which was not certified by peer review) is the author/funder. This article is a US Government work. It is not subject to copyright under 17 USC 105 and is also made available for use under a CC0 license.

Table S8

Cladocopium goreaui-like contaminants identified in GenBank genomes.

Table S9

GenBank genomes with extreme contamination by aggregate contamination length, representing 50% of total contamination identified by FCS-GX.

Table S10

GenBank genomes with extreme contamination by proportion of total sequence length (90-100% contaminated).

Table S11

Summary of genomes with species assignment issues identified by FCS-GX.

Table S12

Comparison of contaminants reported in FCS-GX vs Conterminator.

Table S13

Summary of cleaned RefSeq and RefSeq candidate genomes.

Table S14

Aggregate contamination identified in current RefSeq genomes by FCS-GX, grouped by taxonomic kingdom.

Table S15

FCS-GX chimeric contamination calls in suspected LGT regions.
